# Strand-specific RNA-seq based identification and functional prediction of drought-responsive lncRNAs in cassava

**DOI:** 10.1186/s12864-019-5585-5

**Published:** 2019-03-13

**Authors:** Zehong Ding, Weiwei Tie, Lili Fu, Yan Yan, Guanghua Liu, Wei Yan, Yanan Li, Chunlai Wu, Jiaming Zhang, Wei Hu

**Affiliations:** 1 Key Laboratory of Biology and Genetic Resources of Tropical Crops, Institute of Tropical Bioscience and Biotechnology, Chinese Academy of Tropical Agricultural Sciences, Xueyuan Road 4, Haikou, Hainan China; 2 Institute of Tropical and Sub-tropical Cash Crops, Yunnan Academy of Agricultural Sciences, Baoshan, Yunnan China; 3 Genetic Engineering International Cooperation Base of Chinese Ministry of Science and Technology, Chinese National Center of Plant Gene Research (Wuhan) HUST Part, Key Laboratory of Molecular Biophysics of Chinese Ministry of Education, College of Life Science and Technology, Huazhong University of Science and Technology (HUST), Wuhan, China

**Keywords:** Cassava, lncRNA, PEG treatment, Tissue-specific expression, ssRNA-Seq

## Abstract

**Background:**

Long noncoding RNAs (lncRNAs) have emerged as playing crucial roles in abiotic stress responsive regulation, however, the mechanism of lncRNAs underlying drought-tolerance remains largely unknown in cassava, an important tropical and sub-tropical root crop of remarkable drought tolerance.

**Results:**

In this study, a total of 833 high-confidence lncRNAs, including 652 intergenic and 181 anti-sense lncRNAs, were identified in cassava leaves and root using strand-specific RNA-seq technology, of which 124 were drought-responsive. Trans-regulatory co-expression network revealed that lncRNAs exhibited tissue-specific expression patterns and they preferred to function differently in distinct tissues: e.g., cell-related metabolism, cell wall, and RNA regulation of transcription in folded leaf (FL); degradation of major carbohydrate (CHO) metabolism, calvin cycle and light reaction, light signaling, and tetrapyrrole synthesis in full expanded leaf (FEL); synthesis of major CHO metabolism, nitrogen-metabolism, photosynthesis, and redox in bottom leaf (BL); and hormone metabolism, secondary metabolism, calcium signaling, and abiotic stress in root (RT). In addition, 27 lncRNA-mRNA pairs referred to cis-acting regulation were identified, and these lncRNAs regulated the expression of their neighboring genes mainly through hormone metabolism, RNA regulation of transcription, and signaling of receptor kinase. Besides, 11 lncRNAs were identified acting as putative target mimics of known miRNAs in cassava. Finally, five drought-responsive lncRNAs and 13 co-expressed genes involved in trans-acting, cis-acting, or target mimic regulation were selected and confirmed by qRT-PCR.

**Conclusions:**

These findings provide a comprehensive view of cassava lncRNAs in response to drought stress, which will enable in-depth functional analysis in the future.

**Electronic supplementary material:**

The online version of this article (10.1186/s12864-019-5585-5) contains supplementary material, which is available to authorized users.

## Background

Long noncoding RNAs (lncRNAs) are usually defined as non-protein coding transcripts with > 200 bp in length. According to their genomic origins and their locations relative to nearby protein-coding genes, lncRNA are classified into types of long intergenic noncoding RNAs (lincRNAs), long intronic noncoding RNAs, and long noncoding natural antisense transcripts (lncNATs) [1]. Previously lncRNAs are regarded as transcriptional noises because of their low expression levels, but now emerging evidences have demonstrated that lncRNAs play a crucial role in many plant developmental process including vernalization, reproduction, and photo-morphogenesis [2–4]. In particular, lncRNAs are now considered as important regulatory components in response to abiotic stresses. For examples, *Arabidopsis thaliana* lncRNA *DRIR*, as a novel positive regulator of plant response to drought and salt stress, was involved in ABA signaling, water transport and other stress-relief processes [5]; *npc536* over-expression plants displayed enhanced root growth under salt stress condition compared with wild-type plants [6].

In plants, lncRNAs can execute their functions to respond to stresses in either *cis-acting* or *trans-acting* in the genome via diverse mechanisms, including sequence complementarity or homology with RNAs or DNAs, promoter activity modification by nucleosome repositioning, and epigenetic regulation by DNA methylation and histone modification [1, 7, 8]. Considering the complexity of lncRNA regulation, to date only a few lncRNAs have been functionally characterized in plants, although lncRNAs became more and more attractive in recent years. Recently, target mimic was identified as a regulatory mechanism for lncRNAs to block the interactions between miRNAs and their targets. For examples, *Arabidopsis* phosphate-induced lncRNA *IPS1*, which acts as a target mimic for miR399, can bind and sequester miR399 and reduce miR399-mediated cleavage of *PHO2*, which is involved in phosphate uptake [9]. Similarly, several lncRNAs are found as target mimics for tomato (*Solanum lycopersicum*) miRNAs involved in the infection of tomato yellow leaf curl virus [10].

With the rapid development of high-throughput sequencing technologies, numerous lncRNAs have been identified under drought condition in many species by transcriptome re-assembly. In maize (*Zea mays* L.), a total of 664 drought-responsive lncRNAs were identified, of which 126 were highly similar to known maize lncRNAs while the remaining 538 transcripts were novel lncRNAs [11]. In *Populus trichocarpa*, totally 2542 lncRNA candidates were identified, and 504 out of them were found to be drought responsive [12]. In cotton (*Gossypium hirsutum* L.), a total of 10,820 high-confidence lncRNAs were found under drought and control conditions, of which 9989 were lincRNAs, 153 were intronic lncRNAs, and 678 were anti-sense lncRNAs [13]. However, until now, comprehensive surveys of lncRNAs are still lacking in response to drought stress, especially in tropical crops such as cassava.

Cassava (*Manihot esculenta* Crantz) is one of the important cash crops for many farmers in tropical and sub-tropical regions, and it provides staple food for over 750 million people around the world [14]. Because of its starch-enriched tuberous root, cassava is regarded as a major source for starch production, bio-fuel, and animal feed [15]. Cassava is generally tolerant to drought, however, severe drought stress greatly depresses its growth and development, and finally reduces its economic yield [15]. In the past decades, much progress has been made in the identification and functional characterization of cassava genes and proteins in response to drought stress [16–19]. However, very few studies concerning lncRNAs were performed [20], and the mechanism of lncRNAs underlying cassava drought-tolerance remains largely unknown and therefore needs to be further explored.

In this study, a strand-specific RNA-seq (ssRNA-seq) sequencing approach was applied to investigate the genome-wide transcriptome changes of cassava leaves (at different developmental stages) and root under polyethylene glycol (PEG)-simulated drought condition. Subsequently, drought-responsive lncRNAs were systematically identified, the basic characterization, expression pattern, together with the putative function of these lncRNAs were predicted and analyzed. These findings will expand our knowledge of lncRNAs participating drought response in cassava, and enable in-depth functional analysis of lncRNAs in the future.

## Results

### Drought responses and ssRNA-seq of cassava

Compared with the control (0 h), leaves of cassava seedlings were badly wilted after 24 h of PEG-simulated drought stress (Additional file 1: Figure S1). Similar phenotypes were observed in our previous study [16], which also demonstrated that physiological traits such as peroxidase activity, proline, and soluble protein content were significantly altered, and the expression levels of thousands of genes were dramatically changed after 3 and 24 h of 20% PEG treatment. As an extended research, similar PEG treatments were performed in this study but we mainly focused on the systematic identification and functional characterization of drought-responsive lncRNAs through a ssRNA-seq approach.

**Fig. 1:**
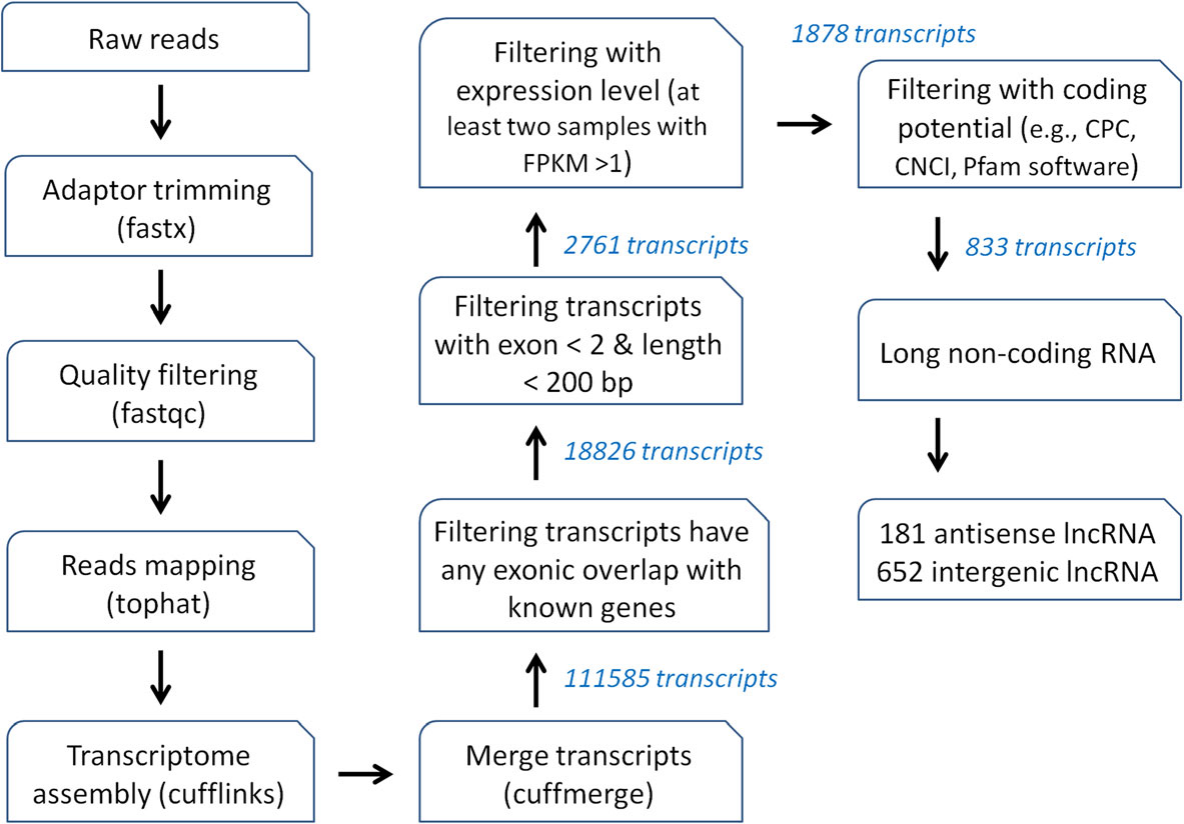
Informatics pipeline for the identification of cassava lncRNAs

After trimming adapters and removing low quality and contaminated reads, in total, 1.49 billion clean reads of 150-bp in length were generated from 24 libraries (12 samples × 2 replicates) by paired-end sequencing with Illumina HiSeq 4000 platform, and ~ 81.3% of them were mapped to the cassava reference genome for further analysis. The total length of all the mapped reads was over 181.8 gigabases (Gb), representing about 351-fold coverage of the cassava genome. Subsequently, a computational pipeline based on ssRNA-seq data was implemented to identify cassava lncRNAs (Fig. 1).

### Identification and characterization of lncRNA in cassava

In total, 111,585 transcripts were obtained after transcriptome re-construction of all ssRNA-seq data using cufflinks pipeline. Subsequently, a few filtering steps were applied to identify the drought-responsive lncRNAs of high-confidence (Fig. 1). Firstly, the transcripts overlapped with known protein-coding genes in the same strand were removed. In this step, a total of 92,759 (~ 83%) transcripts, which were overlapped with 33,033 protein-coding genes representing all annotated genes of the cassava genome, were filtered. Secondly, the transcripts with exon < 2 and length < 200 bp were removed, which resulted in 2761 remained transcripts. Thirdly, the transcripts with FPKM > 1 in less than two samples were removed, to make sure the remaining transcripts were expressed. Next, the transcripts with coding potential, which was evaluated by Coding Potential Calculator (CPC), Coding-Non-Coding Index (CNCI), and the protein families database (Pfam), were removed. Finally, a total of 833 transcripts were obtained, and later they were classified into 652 intergenic and 181 anti-sense lncRNAs according to their genomic locations.

**Fig. 2:**
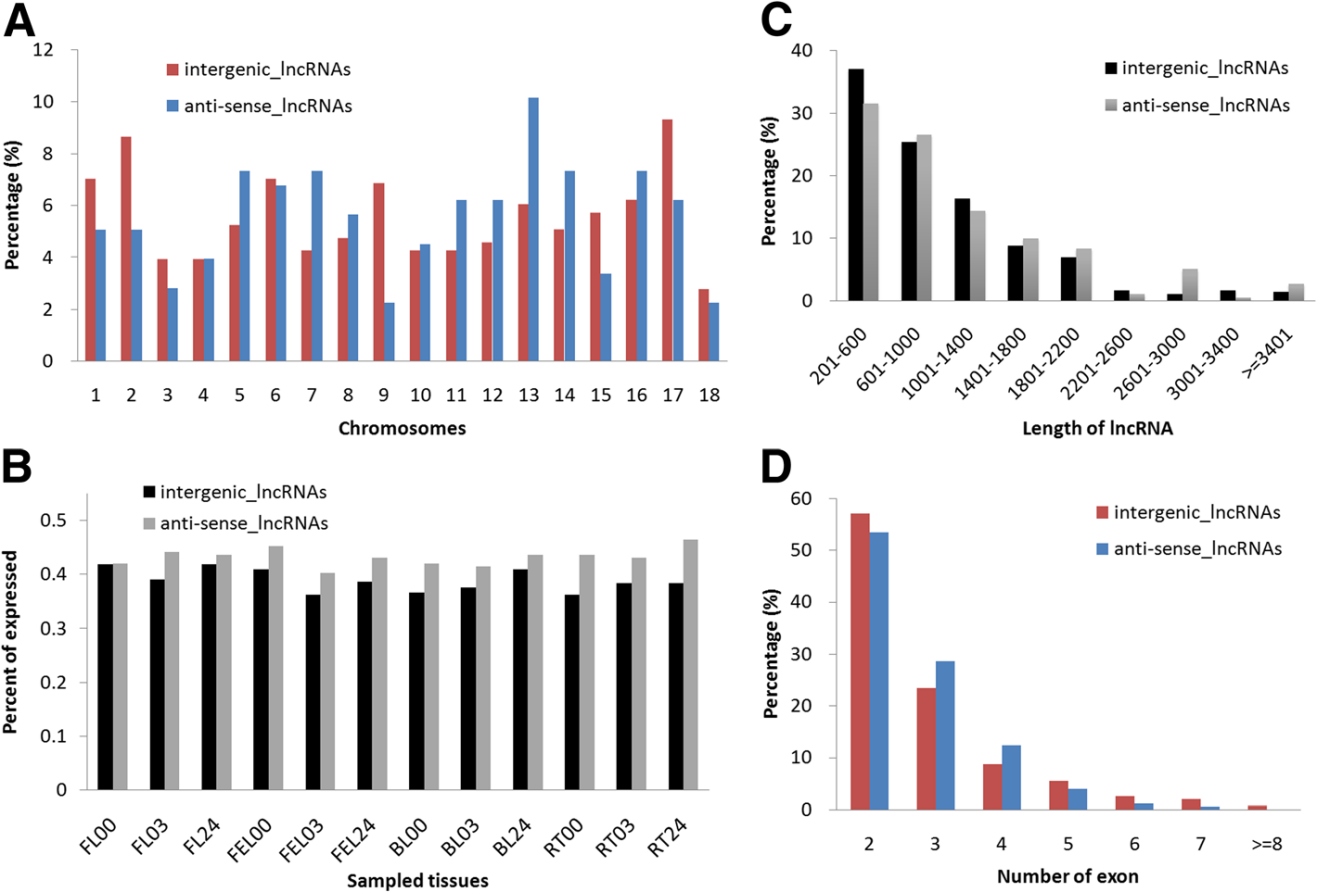
Characterization of cassava lncRNAs under PEG treatments. Distributions of anti-sense and intergenic lncRNAs in **a** chromosome locations, **b** expression levels, **c** transcript length, and **d** exon number, respectively

To characterize the features of these lncRNAs, the distributions of chromosome location, transcript length, exon number, and expression level were evaluated in two groups of intergenic and anti-sense lncRNAs. Overall, intergenic and anti-sense lncRNAs were distributed in all 18 chromosomes of the cassava genome, although different emphases were revealed. For examples, higher percents of intergenic lncRNAs were found in chromosome 1, 2, 9, and 17, while higher percents of anti-sense lncRNAs were found in chromosome 5, 7, 13, and 14 (Fig. 2a). Notably, at the expression level, the percent of expressed anti-sense lncRNAs (FPKM > 1) was higher than that of intergenic lncRNAs in all examined samples (except sample ‘FL00’, Fig. 2b), indicating that anti-sense lncRNAs have a higher probability to be expressed in a given sample compared with intergenic lncRNAs. However, no obvious differences were observed between intergenic and anti-sense lncRNAs regarding the distribution of transcript length and exon number: the median lengths of these intergenic and anti-sense lncRNAs were 787 and 839 nucleotides (nt), respectively, and most of them were shorter than 2200 nt (Fig. 2c); approximately 55% of the cassava intergenic and anti-sense lncRNAs contained two exons, and ~ 26% and ~ 11% contained three and four exons, and only ~ 8% contained at least five exons (Fig. 2d). Together, these results provide a general overview of the characterization of lncRNA in response to drought stress in cassava.

### Identification of differentially expressed (DE) lncRNAs

To explore the transcriptional changes of lncRNAs affected by PEG treatment, DE lncRNAs were identified by pair-wise comparison of samples collected at different time-points within the same tissue, respectively.

**Fig. 3:**
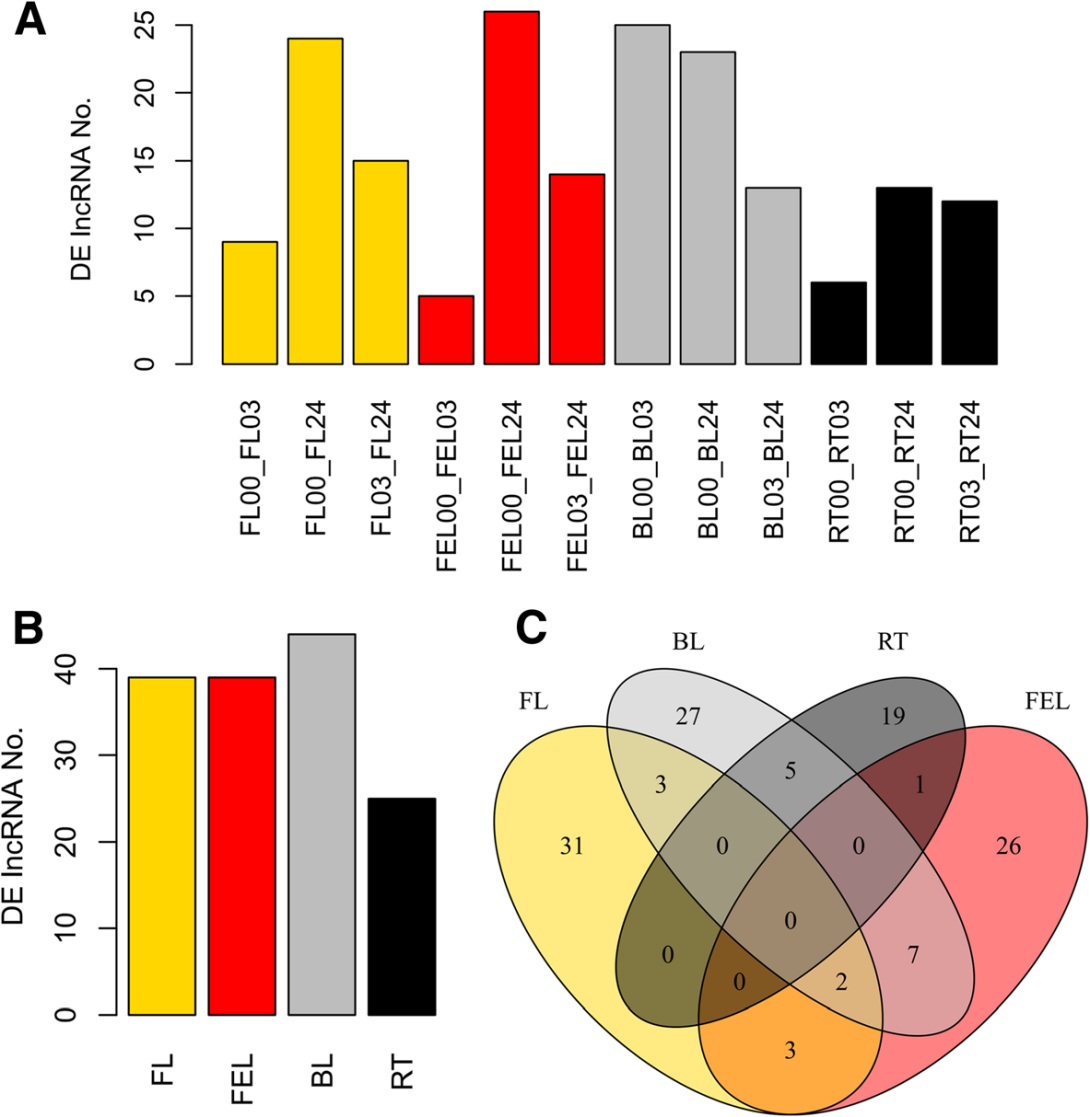
Transcriptome profiling of cassava lncRNAs in response to PEG treatment. Differentially expressed (DE) lncRNAs identified in pair-wise comparison of 12 samples (**a**), four tissues (**b**); and their Venn diagrams (**c**), respectively. FL: folded leaf; FEL: full expanded leaf; BL: bottom leaf; RT: root. The numbers attached behind samples represent the time point at which samples were collected: e.g., 00, 03, and 24 represent 0, 3, and 24 h, respectively

Overall, in FL, FEL, and RT, only a few lncRNAs were differentially expressed at 3 h of PEG treatment, while the numbers of DE lncRNAs were increased more than twice at 24 h. On the contrary, this tendency was clearly different in BL: a great number of lncRNAs were differentially expressed at 3 h upon PEG treatment, whereas only a few lncRNAs were significantly changed at 24 h when compared with 3 h (Fig. 3a). Consistently, a gradient change of DE lncRNA number was observed among different tissues: BL (44) > FEL (39) = FL (39) > RT (25) (Fig. 3b). These results suggested that lncRNAs had a faster and stronger response in old leaf (e.g., BL) than in young leaves (e.g., FEL and FL) and root upon PEG stress.

In total, 124 DE lncRNAs were identified in response to PEG treatment. Most of them were exclusively identified in FL (31), FEL (26), BL (27), and RT (19), and 21 were commonly identified in at least two tissues (Fig. 3c). However, none of lncRNAs were identified in all four tissues. These results indicated that lncRNAs preferred to function in a tissue-specific manner.

### Functional characterization of DE lncRNAs in trans-regulation

To explore the potential functions of drought-responsive lncRNAs, a total of 124 DE lncRNAs, together with 5187 DE genes, were selected and subjected to co-expression analysis to identify trans-regulatory networks of lncRNAs. Subsequently, functional enrichment analysis was performed for the genes of each group (co-expressed module), respectively, and then the enriched functions could be used to predict the functions of lncRNAs that were co-expressed with these DE genes.

**Fig. 4:**
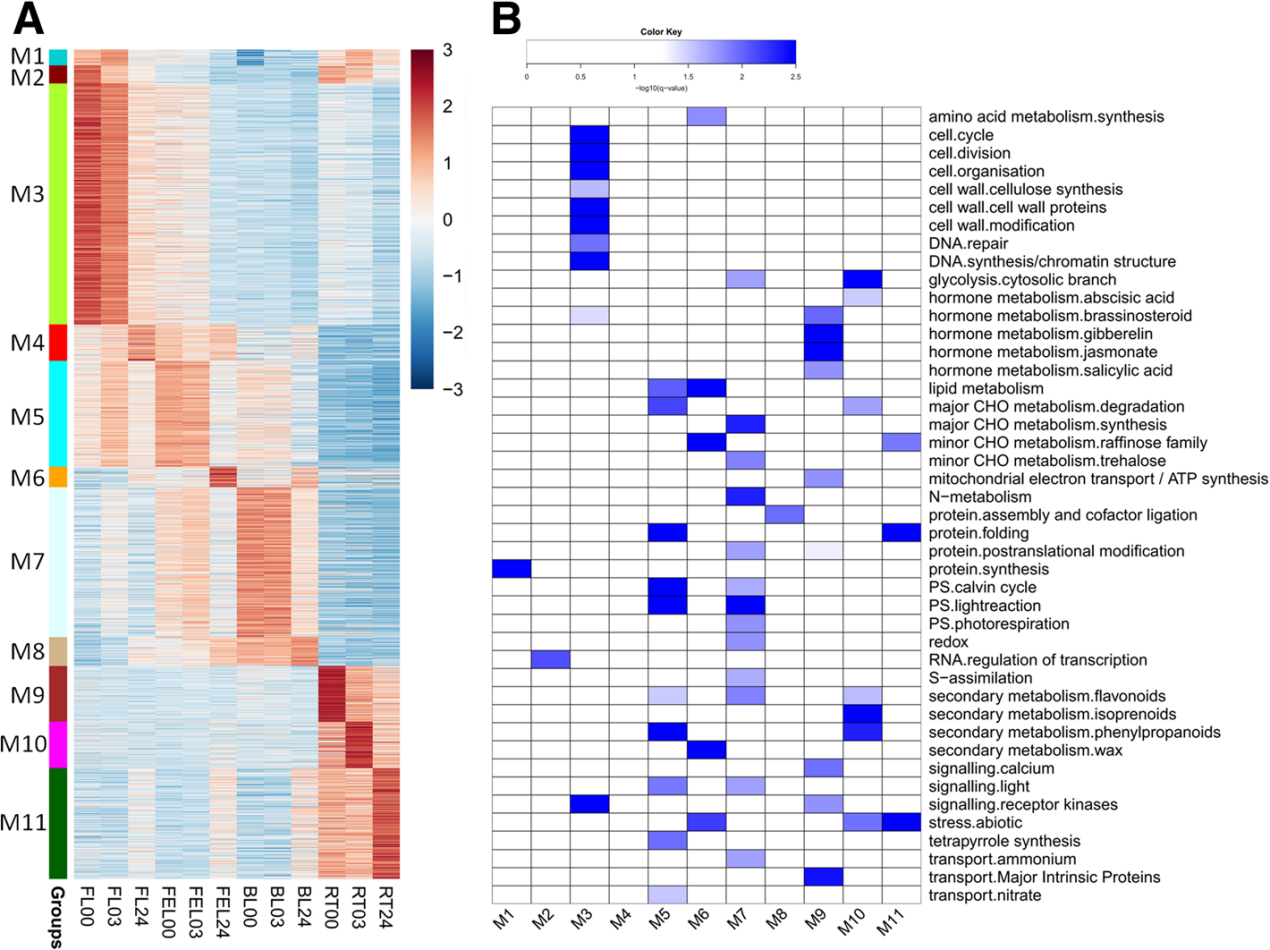
Co-expression analysis of lncRNAs and mRNAs. **a** Heatmap of DE lncRNAs and mRNAs which were mainly clustered into 11 groups (M1-M11). **b** Functional category enrichment of each group presented in (**a**)

In total, 11 groups (M1-M11) were identified based on their different expression patterns (Fig. 4a). The genes/lncRNAs from group M1 to M4 were highly expressed in FL but exhibited different emphases. For examples, group M1 included genes/lncRNAs that were greatly induced at 3 h but decreased at 24 h compared with the expression levels at 0 h, while group M2 included genes/lncRNAs that were gradually decreased at 3 h and 24 h in both FL and RT. These genes from group M1 and M2 were significantly enriched in protein synthesis and RNA regulation of transcription, respectively (Fig. 4b). However, no lncRNAs were included in these two groups. There were 7 lncRNAs in group M3. The genes/lncRNAs included in this group were gradually decreased from 0 h to 24 h in FL, and they were significantly enriched in cell cycle, cell division, cell wall, DNA repair, and signaling of receptor kinases (Fig. 4b). There were 6 lncRNAs in group M4. The genes/lncRNAs from this group were gradually induced from 0 h to 24 h in FL upon PEG treatment, however, no enriched categories were found in this group.

The genes/lncRNAs from group M5 to M6 were highly expressed in FEL (Fig. 4a). The expression of the former group was greatly suppressed, while that of the latter group was dramatically induced at 24 h of PEG treatment. There were 5 lncRNAs in group M5, of which the genes were significantly enriched in lipid metabolism, degradation of major carbohydrate (CHO) metabolism, calvin cycle and light reaction, secondary metabolism, light signaling, and tetrapyrrole synthesis. The genes included in group M6 were significantly enriched in amino acid synthesis, lipid metabolism, secondary metabolism of wax, and abiotic stress, but none of lncRNAs were included in this group (Fig. 4b).

The genes/lncRNAs from group M7 to M8 were highly expressed in BL (Fig. 4a). Similar to group M5 and M6, the former group was greatly decreased whereas the latter group was significantly increased at 24 h of PEG treatment. There were 13 lncRNAs in group M7, of which the enriched categories included synthesis of major CHO metabolism, trehalose metabolism, nitrogen (N)-metabolism, photosynthesis, redox, secondary metabolism of flavonoids, and light signaling. There was only one lncRNA in group M8, and this group was significantly enriched in protein assembly and cofactor ligation (Fig. 4b).

The genes/lncRNAs from group M9 to M11 were highly expressed in RT (Fig. 4a). It was clearly observed that the expression was dramatically decreased from 0 h to 24 h upon PEG treatment in group M9, which contained only one lncRNA. The enriched categories in this group included hormone metabolisms such as gibberellin and jasmonate, mitochondrial electron transport/ATP synthesis, calcium signaling, and receptor kinases signaling. There were 4 and 9 lncRNAs in group M10 and M11, respectively. The genes/lncRNAs from group M10 were greatly induced at 3 h but suppressed at 24 h, and they were significantly enriched in abscisic acid (ABA), degradation of major CHO metabolism, secondary metabolisms, and abiotic stress. On the contrary, the genes/lncRNAs from group M11 were dramatically induced at 24 h after PEG treatment, and they were significantly enriched in raffinose metabolism, protein folding, and abiotic stress (Fig. 4b).

Taken together, these results revealed that the genes/lncRNAs were exhibited in a tissue-specific manner in response to PEG treatment in cassava, and also suggested that the genes/lncRNAs preferred to function differently in distinct tissues: e.g., cell-related metabolism, cell wall, RNA regulation of transcription in FL; degradation of major CHO metabolism, calvin cycle and light reaction, light signaling, and tetrapyrrole synthesis in FEL; synthesis of major CHO metabolism, N-metabolism, photosynthesis, and redox in BL; and hormone metabolism, secondary metabolism, calcium signaling, and abiotic stress in RT.

### Functional characterization of DE lncRNAs in cis-regulation

To further explore the potential functions of drought-responsive DE lncRNAs, protein-coding genes, which were spaced 10 k/100 k upstream and downstream of these lncRNAs, were selected and subjected to co-expression analysis. The lncRNA-mRNA pairs that were highly correlated and closely located were specifically attractive in a cis-acting regulatory relationship.

**Fig. 5:**
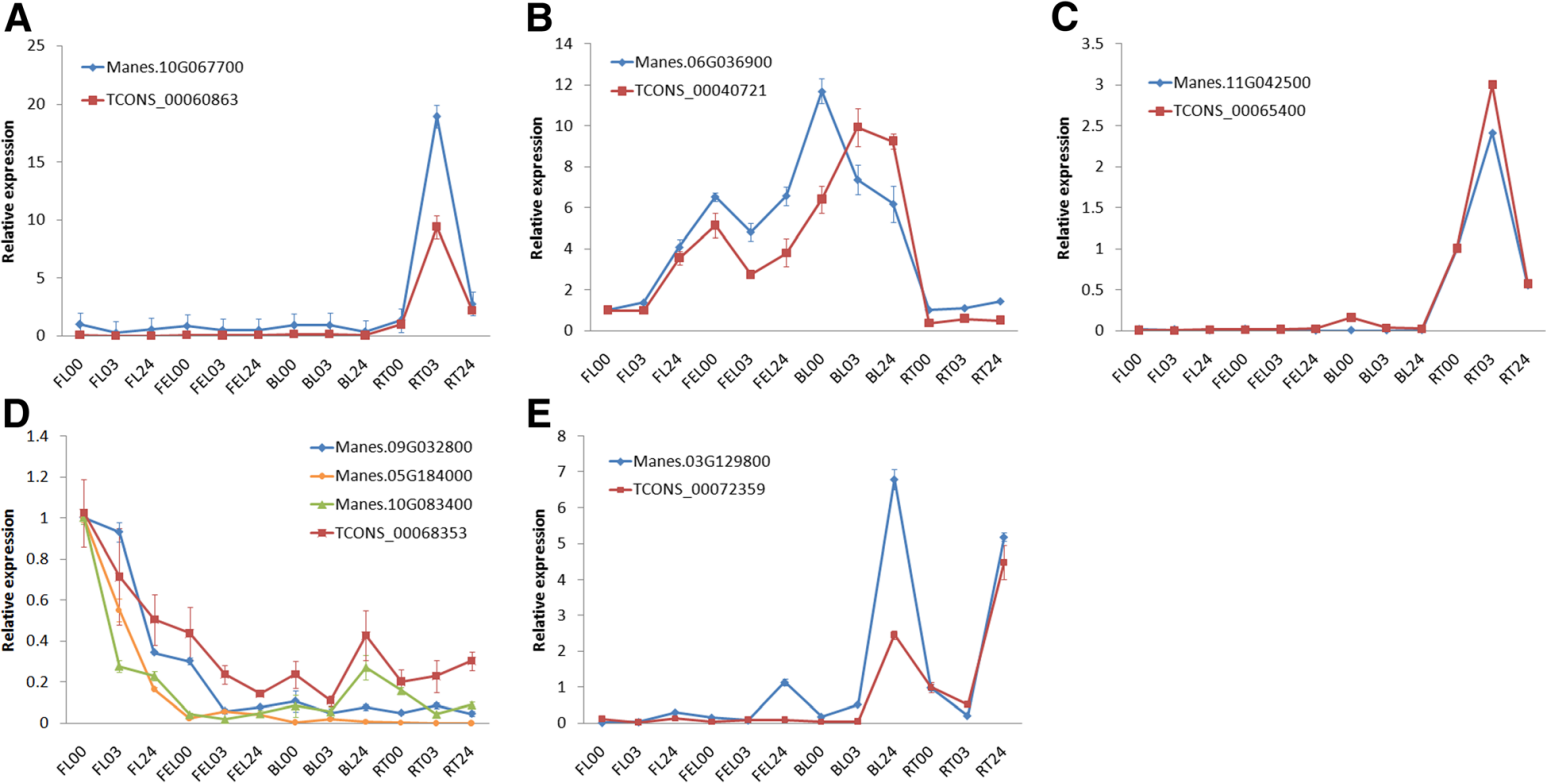
Confirmation of the expression patterns of lncRNAs and their associated genes by qRT-PCR. **a**-**c** the lncRNA-mRNA pairs involved in cis-acting regulation; **d**, **e** lncRNAs and the related genes involved in target mimic regulation. The values are shown as mean ± standard deviation of three independent replicates

In total, 27 lncRNA-mRNA pairs involved in cis-acting regulation were identified (Additional file 2: Table S1). Of which, TCONS_00033864 was located 3798 bp upstream of Manes.05G018500 encoding a SAUR-like auxin-responsive gene, TCONS_00060863 was located 2447 bp downstream of Manes.10G067700 encoding 8-hydroxylase involved in ABA catabolism (Fig. 5a), TCONS_00097416 was located 6655 bp upstream of Manes.16G102700 involved in ethylene signaling, suggesting these lncRNAs were involved in hormone regulation in response to drought stress. TCONS_00040721 was spaced 6652 bp upstream of Manes.06G036900 encoding an AP2/EREBP transcription factor (Fig. 5b), and TCONS_00069665 was spaced 91,056 bp upstream of Manes.11G134100 encoding a C2H2 zinc finger transcription factor, indicating that these two lncRNAs participated in RNA regulation of transcription. In addition, several lncRNAs involved in signaling of receptor kinase were also found, e.g., TCONS_00065400 was located 37,640 bp upstream of Manes.11G042500 encoding a member of proline-rich extensin-like receptor kinase (Fig. 5c), and TCONS_00099153 was spaced 5973 bp upstream of Manes.17G056800 encoded a leucine-rich repeat protein kinase and required for root hair elongation.

Together, these results suggested that these DE lncRNAs, which might act as regulators in cis-acting in response to PEG treatment, regulated the expression of their neighboring genes mainly through hormone metabolism, RNA regulation of transcription, and signaling of receptor kinase.

### Functional prediction of lncRNAs acting as miRNA target mimics

lncRNAs have been demonstrated to function through miRNAs for transcriptional, post-transcriptional, and epigenetic gene regulation, therefore, it’s of great importance to investigate the crosstalk between lncRNAs and miRNAs by exploring the lncRNAs acting as target mimic of known miRNAs in cassava.

In total, 11 lncRNAs were identified acting as target mimics of known miRNAs, such as miR156, miR164, miR169, and miR172 (Additional file 3: Table S2). miR156 is stress-induced and it targets SPL genes (e.g., *SPL9*) in plant development and abiotic stress tolerance [21, 22]. As a target mimic of miR156k, TCONS_00068353 was greatly suppressed in FL, and consistently, a homolog of *SPL9* (Manes.09G032800) exhibited similar expression trend of TCONS_00068353 under PEG treatment (Fig. 5d and Additional file 3: Table S2). miR172 participated in water deficit and salt stress through the expression regulation of AP2-like transcription factors [23], and its expression was promoted by *SPL* genes [24]. Further studies revealed that SPL/miR156 module can interact with the AP2/miR172 unit in barely [25]. It’s worthy to note that, in our study, TCONS_00068353 was bound with miR172c, coordinated with the decreased expression of miR172-targeted AP2-like gene (Manes.05G184000) in FL under PEG treatment. In addition, TCONS_00068353/Manes.09G032800 and TCONS_00068353/Manes.05G184000 showed similar expression patterns in response to PEG treatment, supporting the interactions between SPL/miR156 module and AP2/miR172 unit [25].

Besides the miRNA-mRNA interactions consistent with the previously reporters, some different and currently unknown interactions were identified. For examples, *MYC2* and *CSD2* were the targets of miR169 and miR398, respectively, but they were predicted as the targets of miR164a and miR171g in our study, in accordance with the similar expression patterns of TCONS_00068353 and TCONS_00072359 (Fig. 5e and Additional file 3: Table S2) which acted as the target mimics of miR164a and miR171g, respectively.

Together, these results strongly suggested that lncRNAs might function through miRNAs in the response of drought stress in cassava.

### Validation of lncRNAs and genes by qRT-PCR

**Fig. 6:**
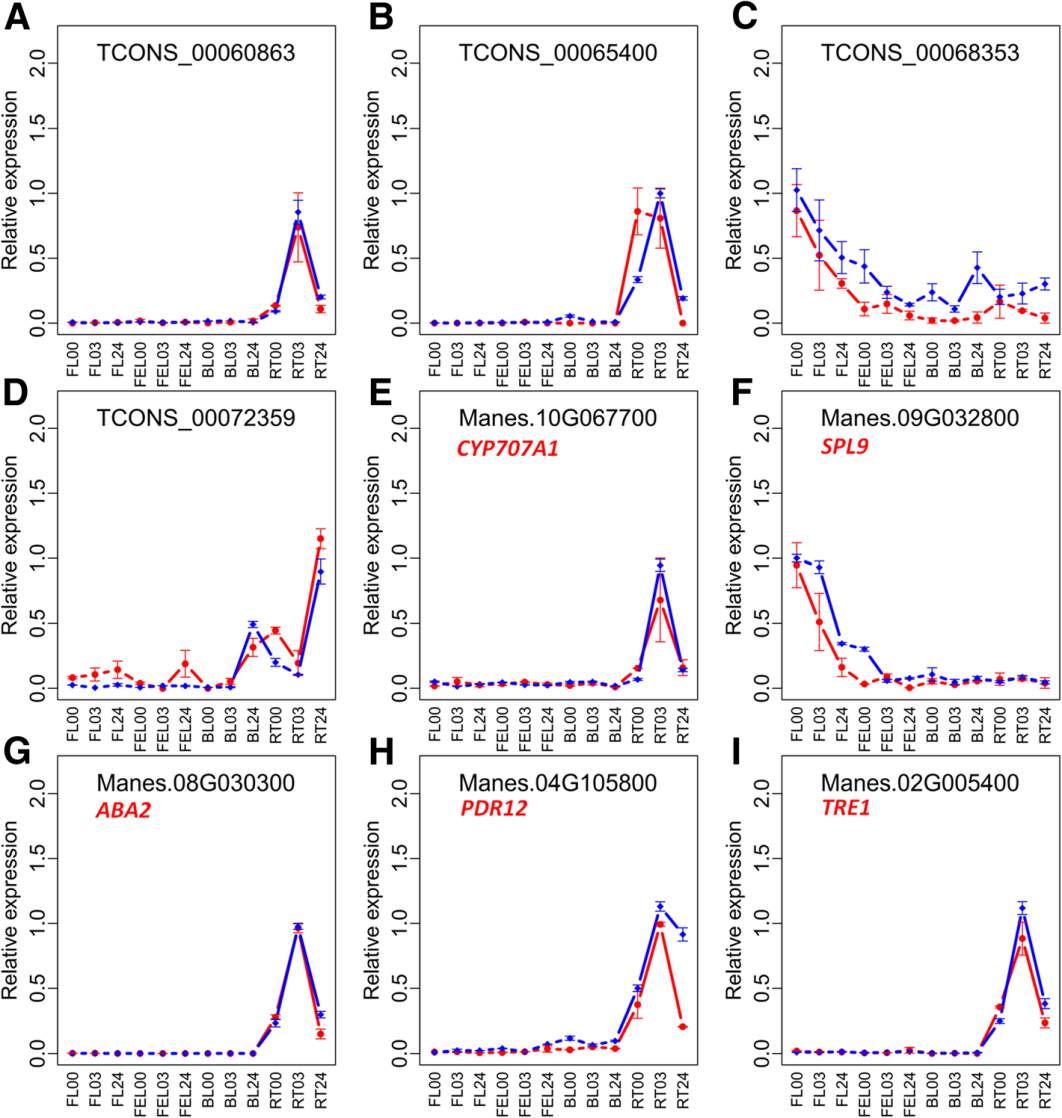
Expression validation of selected lncRNAs and genes between ssRNA-seq and qRT-PCR. **a-d** relative expression of lncRNAs; **e-i** relative expression of genes. Expression values are normalized by the maximum value among samples of each lncRNA/gene and presented as mean ± standard deviation of two and three independent replicates from ssRNA-seq (red lines) and qRT-PCR (blue lines), respectively

To validate the expression results of ssRNA-seq data, a total of five crucial lncRNAs, which were involved in trans-acting, cis-acting, or miRNA target mimics, and 13 co-expressed genes were tested by qRT-PCR method. Overall, high correlation coefficients (R = 0.78–0.99) were revealed between these two independent measurements (Fig. 6 and Additional file 4: Table S3), indicating that the expression patterns of lncRNAs and genes based on ssRNA-seq data are reliable.

## Discussion

### lncRNA is a key player in cassava drought stress

lncRNAs have well demonstrated to play essential roles in drought stress response in many plants, including *Arabidopsis* [26], rice (*Oryza sativa*) [27], maize [11], cotton [13], foxtail millet (*Setaria italica*) [28], and *Populus* [12]. In contrast, very a few lncRNAs were comprehensively identified in tropical species, especially in cassava, a tropical plant with outstanding tolerance to drought stress. In this study, a total of 833 lncRNAs, including 652 intergenic lncRNAs and 181 anti-sense lncRNAs, were identified in cassava using ssRNA-seq strategy. The number of lncRNAs was far less than that identified in cotton and *Populus* [12, 13], but more than that identified in foxtail millet and maize [11, 28]. This number was also 1.2-fold higher than that identified recently in cassava [20], even more strict criteria were applied in our study. These results, together, suggested that the number of lncRNAs identified by sequencing might depend largely on the species, sequencing depth, and the criteria of lncRNAs identification.

Similar to the characteristics reported previously [20], the majority of cassava lncRNAs contained 2–3 exons, however, the median length was much shorter in our case. Besides, it seems that intergenic and anti-sense lncRNAs preferred to locate on certain chromosomes, respectively (Fig. 2a). In addition to the basic characterizations, we also compared our lncRNAs with that identified previously [20] and found that only 57 (~ 6.5%) lncRNAs were commonly identified, thus the remaining 776 can be regarded as novel cassava lncRNAs identified in our study. Further inspection revealed that ~ 28.5% (221/776) lncRNAs were not expressed (FPKM < 1) in both FL and FEL samples, which might be one of the explanations for why these lncRNAs were not previously identified.

lncRNAs were reported to exhibit organ-specific or tissue-specific expression patterns in regulating response to abiotic stress such as drought [11, 29]. In our study, totally 124 DE lncRNAs were found, and most (~ 83%) of them were exclusively identified in only one tissue (Fig. 3c). Further analysis revealed that 46 lncRNAs, together with thousands of co-expressed DE genes, were clustered into a total of 11 groups with diverse expression patterns along different time-points of PEG treatment in four tissues (Fig. 4a). Consistent to the results previously reported in other plants [11, 29], our findings strongly indicating that cassava lncRNAs were tissue-specifically expressed under drought condition and they might play different functions in distinct tissues as revealed by functional enrichment assay (Fig. 4b).

### Functional prediction of cassava lncRNAs in response to drought stress

Emerging evidences have demonstrated that lncRNAs can act *in trans* to regulate the expression level of multiple genes located throughout the genome [20, 30], therefore, in this study, a co-expression network analysis was performed to predict the functions of lncRNAs according to the functional enrichment of co-expressed DE genes. Consistent to our previous study [16], genes involved in cell cycle and cell organization, cell wall, calvin cycle and light reaction, major CHO metabolism, secondary metabolism, signaling receptor kinase, hormone metabolism (such as ABA and GA), and abiotic stress were significantly enriched. Notably, this result was consistently obtained from two independent RNA-seq experiments, strongly suggested that lncRNAs were involved in these similar functions of their co-expressed genes under drought stress in cassava. Comparable functions of lncRNAs were also reported in other species. In cotton, Lu et al. [13] concluded that lncRNAs were likely to be involved in hormone signal transduction, carbon fixation of photosynthesis, secondary metabolism, and RNA transport in response to drought stress; in *Arabidopsis*, lncRNA *DRIR* was significantly activated by drought and salt stress, and it participated in the expression regulation of genes involved in ABA signaling, water transport, and transcription [5]; in cassava, Li et al. [20] found that lncRNAs were mainly associated with hormone signal transduction, starch and sucrose metabolism, and secondary metabolic pathways, and suggested that transcriptional regulation of gene expression might be one of the principal roles of lncRNAs in response to drought and/or cold stresses.

Besides, lncRNAs also can act *in cis* to regulate the expression of their neighboring genes. Specifically, in maize, lncRNA *Vgt1* influenced the expression of *ZmRap2* which located as far as ~ 70 kb downstream of *Vgt1* [31]. In this study, a total of 27 lncRNA-mRNA pairs involved in *cis-acting* regulation were identified. The adjacent genes influenced by these lncRNAs were mainly involved in hormone metabolism, RNA regulation of transcription, and signaling of receptor kinase. For examples, TCONS_00060863 was located 2447 bp downstream of Manes.10G067700 encoding 8-hydroxylase involved in ABA catabolism (Fig. 5a), TCONS_00040721 was spaced 6652 bp upstream of Manes.06G036900 encoding an AP2/EREBP transcription factor (Fig. 5b), and the expression levels of these lncRNA-mRNA pairs were further verified by qRT-PCR (Additional file 4: Table S3).

### Networks of lncRNAs, miRNAs, and mRNAs involved in drought response of cassava

**Fig. 7:**
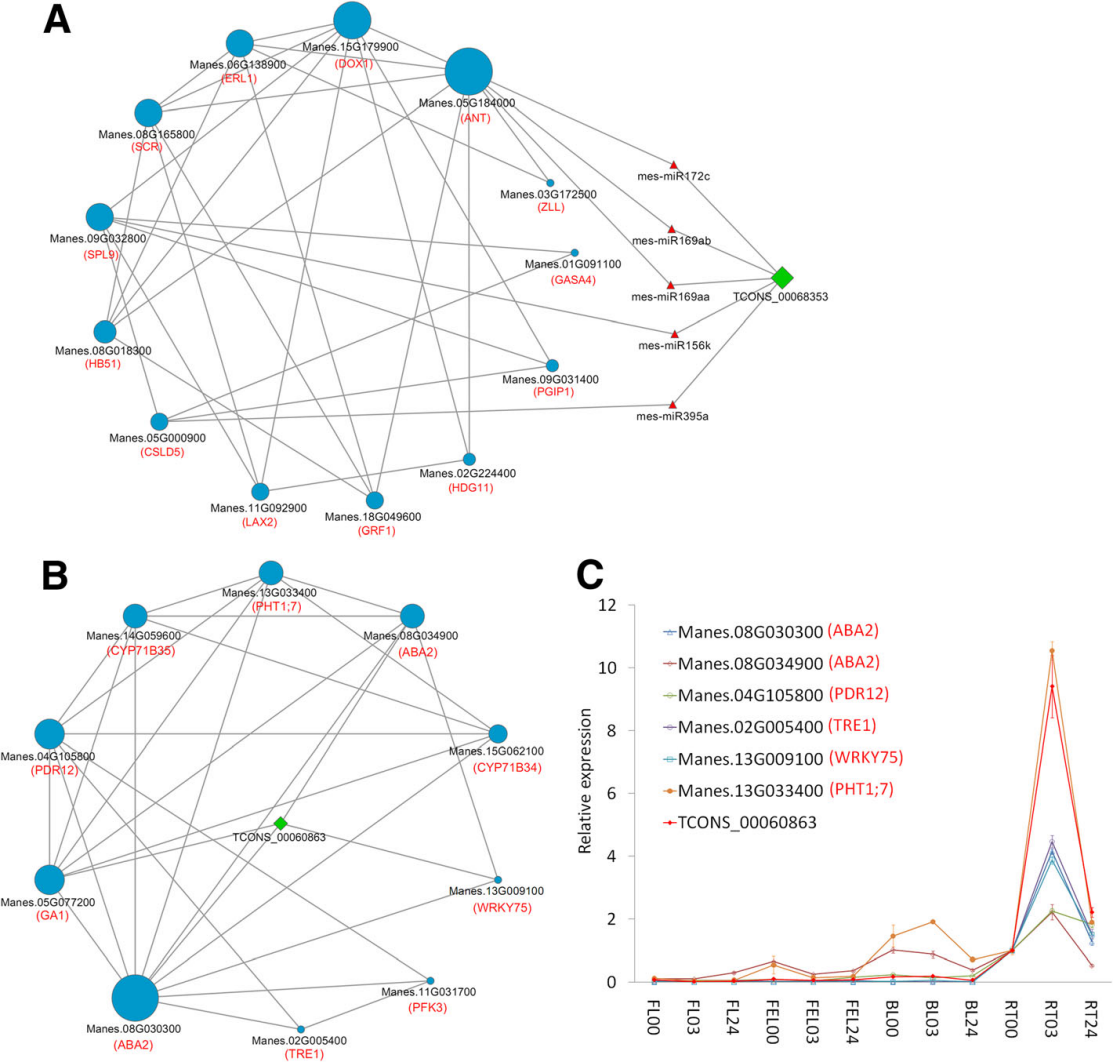
Representative networks of lncRNAs, miRNAs, and mRNAs. **a** A representative network of lncRNAs, miRNAs, and mRNAs involved in target mimic regulation. **b** A representative network of lncRNAs and mRNAs involved in ABA signaling. Genes, lncRNAs, and miRNAs were represented by blue cycles, green diamonds, and red triangles, respectively, and their sizes were determined according to their degrees/connections to others. The gene names were in red in the brackets. **c** Expression validation of selected lncRNAs and genes in (**b**) by qRT-PCR. The values are shown as mean ± standard deviation of three independent replicates

In addition to trans- and cis-acting regulation, lncRNAs also can function as miRNA target mimic to regulate the expression of multiple genes [9, 32]. Take *Arabidopsis* lncRNA *IPS1* for an example, it acts as a target mimic for miR-399, therefore, *IPS1* over-expression causes increased mRNA accumulation of miR-399 target gene *PHO2*, which is involved in Pi homeostasis [9]. In this study, a total of 11 lncRNAs were predicted as target mimic of 24 miRNAs, of which miR156, miR169, miR172, and miR395 were well characterized to be involved in abiotic stress [7, 33]. For examples, miR156 and miR172 respectively target *SPL* and *AP2* genes in plant development and abiotic stress such as drought and salt [21, 23]. Moreover, SPL/miR156 module can interact with AP2/miR172 unit in various biological processes [25]. In our case, it worthy to note that TCONS_00068353, which was predicted as a target mimic of miR156k and miR172c, exhibited consistent expression pattern of homologs of *SPL9* (Manes.09G032800) and an AP2-like gene (Manes.05G184000). In addition, TCONS_00068353 was co-expressed with many genes involved in plant growth (*CSLD5*), cell division and organization (*ERL1*, *SPCH*, and *LAX2*), trichome branching (*HDG11*), root development (*SCR*), leaf development (*GRF1* and *HB51*), and abiotic stress (*DOX1*, Fig. 7a and Additional file 5: Table S4). Together, these results strongly suggest that TCONS_00068353 is a key candidate acting as target mimic of miR156k and miR172c to regulate the expression of genes involved in plant growth and development and abiotic stress, in accordance with the roles of miR156 and miR172 reported in other plants [21, 23].

ABA is a key hormone involved in the response of plant biotic and abiotic stress [34]. To investigate the changes of ABA levels in different tissues and at different time-points of drought treatment, ABA contents were determined in our samples, respectively, and the results showed that ABA levels were significantly increased in BL and RT at 3 h and 24 h whereas the levels were almost unchanged in FL and FEL during the drought treatment (Additional file 6: Figure S2), suggesting that ABA functions mainly in BL and RT of cassava under drought stress. Accordingly, *HAB1*, a negative regulator of ABA signaling, was greatly suppressed in both BL and RT under PEG treatment. On the contrary, *NCED9*, a key gene involved in ABA biosynthesis, was greatly suppressed in BL and RT upon PEG treatment, indicating a possible negative feedback regulation of ABA biosynthesis as previously described [35]. In this work, genes related to ABA pathways were significantly enriched in group G10 and their expression levels were dramatically altered in root, consistent with our previous study [16]. Of which, Manes.08G030300 encoded an ABA DEFICIENT 2 (*ABA2*) gene involved in the conversion of xanthoxin to ABA-aldehyde during ABA biosynthesis, and it was a key hub gene with most connections to other genes in this group. To explore the ABA-involved networks in response to drought, this gene and its most connected genes were selected and visualized. As shown in Fig. 7b, an ABA transporter *PDR12* (Manes.04G105800), which is a homolog of *AtPDR12* that is necessary for timely responses to ABA under drought and involved in ABA-regulated lateral root development [36], and another *ABA2* gene (Manes.08G034900) directly related to ABA pathways were included in this network. Notably, these genes were co-expressed with TCONS_00060863, which was also found to regulate *CYP707A1* (Manes.10G067700) encoding 8-hydroxylase involved in ABA catabolism in cis-acting (Fig. 5a), strongly indicating that TCONS_00060863 was a key lncRNA involved in ABA signaling pathway under drought condition. In addition, a homolog of *AtTRE1*, which was greatly induced by ABA treatment and involved in drought stress tolerance [37], was also included. Compared with wild-type plants, *AtTRE1* over-expressing lines showed enhanced root growth on trehalose-containing medium [37], indicating its possible roles for root development in drought stress. WRKY transcription factors (TFs) are key components in ABA signaling [38], of which *WRKY75* is well characterized in phosphate (Pi) stress response and root development, and it can activate several Pi starvation-induced genes encoding phosphatases, *Mt4/TPS1*-like genes, and high-affinity Pi transporters [39]. Recently, *WRKY75* is also known as a novel component of gibberelin (GA)-mediated signaling pathway [40]. Interesting, a homolog of *WRKY75*, together with *GA1* involved in GA biosynthesis and *PHT1;7* related to Pi transport and specifically induced in Pi-deprived roots [41], were included in this network (Additional file 7: Table S5), suggesting that *WRKY75* might also be involved in ABA signaling under drought stress in cassava. Consistently, *CiWRKY75*, which showed the highest sequence similarity to *AtWRKY75* of *Arabidopsis* WRKY family, was significantly induced by salt and ABA treatment in *Caragana intermedia* [42]. In addition, several genes related to oxidation reduction, e.g., *CYP71B34* and *CYP71B35*, were also included. Together, these results revealed a complex network of ABA signaling in drought response of cassava, and suggested that a possible role of this network is responsible for root development under stress conditions, as indicated by a few functionally well-characterized genes such as *PDR12*, *TRE1*, and *WRKY75* [36, 37, 39].

## Conclusions

In this study, a large number of drought-responsive lncRNAs were systematically identified in cassava leaves and root, their basic characterizations were investigated, and their potential functions were predicted via trans-acting, cis-acting, and miRNA target mimics. These findings provide a comprehensive view of cassava lncRNAs in response to drought stress and expand our knowledge of lncRNAs in the signaling regulatory networks under drought condition, which will enable in-depth functional analysis in the future.

## Methods

### Plant materials and treatments

This experiment was conducted as previously described [16]: the stems of cassava variety, Ku50, were cut into ~ 15 cm in length with two to three buds and planted vertically in pots (height × bottom diameter × upper diameter = 18.8 cm × 14.8 cm × 18.5 cm) with soil and vermiculite (1:1) in the glass house in the Chinese Academy of Tropical Agricultural Sciences, Haikou, China. Forty-five days later, uniform cassava seedlings were chosen and subjected to drought stress simulated by using 20% PEG 6000 solution according to our previous study [16]. Different developmental leaves, including folded leaf (FL), full expanded leaf (FEL) and bottom leaf (BL), as well as root (RT) were collected at 0, 3 and 24 h after PEG treatment and frozen immediately in liquid nitrogen. Each sample was pooled from five plants with three replicates. Subsequently, two replicates of these samples were chosen for ssRNA-seq sequencing instead of regular RNA-seq used previously [16]. For each sample, ABA contents were determined by using plant enzyme-linked immunosorbent assay (ELISA) kits (MeiLian Biotechnology, Shanghai, China) with triple replicates, respectively.

### RNA extraction, library construction and sequencing

The total RNA extraction, transcriptome libraries preparation, and ssRNA-seq sequencing were conducted by the Annoroad Gene Technology Corporation (Beijing, China). Briefly, the integrity and quality of total RNA were examined by a Nanodrop ND-2000 spectrophotometer (Thermo Scientific Inc., USA) and an Agilent 2100 Bioanalyzer (Agilent, USA). The RNA-seq libraries were constructed using Illumina TruSeq™ RNA sample prep Kit (Illumina, San Diego, CA, USA) with Ribo-Zero Magnetic kit for rRNA depletion according to the manufacturer’s instructions, and subsequently sequenced on the Illumina Hiseq 4000 platform with 150 bp paired-end reads.

### Identification of lncRNAs

After trimming the adaptor sequences and removing low-quality reads, clean reads were obtained and mapped to the cassava reference genome using Tophat 2.0 [43] with ‘-library-type fr-firststrand’ parameters. Subsequently, Cufflinks pipeline [44] was employed to assemble reads into transcripts, and the assembled transcripts found in at least two samples were chosen for further analysis. The expression levels were calculated as fragments per kilobase per million mapped reads (FPKM). For identification of lncRNA, a three-step pipeline was adopted [20] and used: 1) the transcripts that overlapped with known protein-coding genes on the same strand, that with length < 200 bp, that with exon number < 2, that with ORF length > 300, and that with minimal reads coverage < 3 were removed; 2) the transcripts with coding potential were removed based on the evaluation of Coding Potential Calculator (CPC) [45], Coding-Potential Assessment Tool (CPAT) [46], and Coding-Non-Coding Index (CNCI) [47]; 3) the transcripts with known protein domains were also excluded according to Pfam-hidden Markov models (HMMs) [48]. The remaining transcripts were considered as reliable lncRNAs. Differentially expressed (DE) lncRNAs were pair-wisely identified setting false discovery rate < 0.05 and |log2fold-change| > 1.

### Prediction of lncRNA target and functional enrichment

For identification of target genes in trans-regulation, DE lncRNAs, together with DE genes, were subjected to the standard procedure of WGCNA [49]. The lncRNAs and genes within the same group (module) were of similar expression patterns and potentially in trans-regulation. To predict the function of lncRNAs in trans-regulation, cassava loci were functionally annotated and classified into hierarchical categories based on MapMan [50], and the significantly over-represented functional categories were determined according to the Fisher’s exact test as previously reported [18, 51].

For identification of target genes in cis-regulation, protein-coding genes, which were spaced 10 k/100 k upstream and downstream of lncRNAs, were selected and also subjected to co-expression analysis. The lncRNA-mRNA pairs that were co-expressed and closely located were in cis-acting regulatory relationships.

### Prediction of lncRNAs acting as miRNA target mimics

Target mimics were predicted by submitting all of the discovered DE lncRNAs and the cassava miRNAs (miRBase Release 22, March 2018) to psRNATarget [52], with less than four mismatches and G/U pairs allowed within the lncRNA-miRNA pairing regions, according to the principles established by Wu et al. [32].

### Quantitative RT-PCR (qRT-PCR) analysis

Total RNA was isolated from each sample using RNAiso reagent (OMEGA), respectively, and reverse transcription of the first-strand cDNA was performed by PrimeScript™ RT reagent Kit with gDNA Eraser (TaKaRa, Dalian, China). To validate the results of ssRNA-seq, a total of five DE lncRNAs, together with 13 co-expressed genes, were selected and confirmed by qRT-PCR with primers listed in Additional file 4: Table S3. The qRT-PCR was performed using SYBR Premix Ex Taq™ (TaKaRa, Dalian, China) on a Stratagene Mx3000P machine (Stratagene, CA, USA), and the conditions were as follows: 30 s at 95 °C; followed by 40 cycles of 10 s at 95 °C and 30 s at 60 °C. Then, a thermal denaturing step generating the melt curves was followed to verify the amplification specificity. The cassava *actin* gene was used as the endogenous control [16]. Each sample was measured in triplicate, and the relative expression levels were calculated using the 2^-ΔΔCt^ method.

## Additional files

Additional file 1: **Figure S1.** Phenotypes of cassava under PEG-simulated drought stress. After 24 h of PEG treatment, leaves were badly wilted as indicated by a red box. (TIF 6252 kb)

Additional file 2: **Table S1.** Summary of 27 lncRNA-mRNA pairs in cis-acting regulatory relationship. (XLSX 23 kb)

Additional file 3: **Table S2.** Prediction of lncRNAs acting as miRNA target mimics. (XLSX 30 kb)

Additional file 4: **Table S3.** Primers of lncRNAs and genes used for qRT-PCR and the expression correlation between ssRNA-seq and qRT-PCR. (XLSX 15 kb)

Additional file 5: **Table S4.** Summary of genes involved in mRNA-lncRNA-miRNA network presented in Fig. 7a. (XLSX 15 kb)

Additional file 6: **Figure S2.** ABA content determined in leaves and roots under drought. Data were shown as mean ± standard deviation derived from three biological replicates, and values with different letters were significant (*P* < 0.05) based on Duncan’s multiple range tests. (TIF 55 kb)

Additional file 7: **Table S5.** Summary of genes involved in mRNA-lncRNA network presented in Fig. 7b. (XLSX 13 kb)

## Abbreviations

ABA: Abscisic acid
BL: Bottom leaf
CHO: Carbohydrate
CNCI: Coding-Non-Coding Index
CPAT: Coding-Potential Assessment Tool
CPC: Coding Potential Calculator
DE: Differentially expressed
FEL: Full expanded leaf
FL: Folded leaf
FPKM: Fragments per kilobase per million mapped reads
GA: Gibberelin
Gb: gigabases
HMMs: Hidden Markov models
lincRNAs: Long intergenic noncoding RNAs
lncNATs: Long noncoding natural antisense transcripts
lncRNAs: Long noncoding RNAs
N: Nitrogen
nt: Nucleotides
PEG: Polyethylene glycol
Pfam: The protein families database
Pi: Phosphate
qRT-PCR: quantitative RT-PCR
RT: Root
ssRNA-seq: Strand-specific RNA-seq
TFs: Transcription factors
