# Supplementary figures and images for: Strand-specific RNA-seq based identification and functional prediction of drought-responsive lncRNAs in cassava
Source: BMC Genomics. 2019 Mar 13;20:214. doi: 10.1186/s12864-019-5585-5 (PMC6417064; doi:10.1186/s12864-019-5585-5)

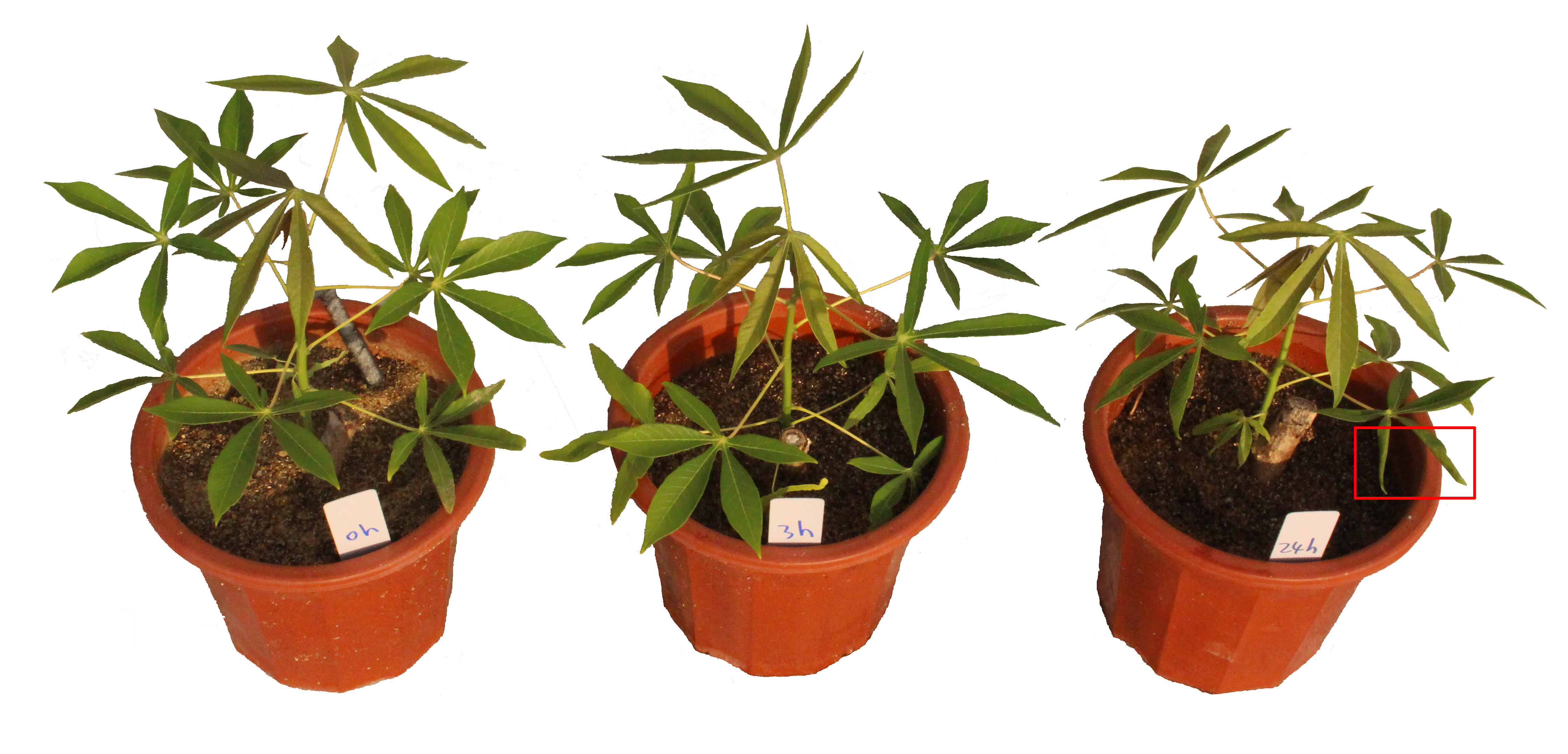

Supplement: Supplementary file 1 — Figure S1. Phenotypes of cassava under PEG-simulated drought stress. After 24 h of PEG treatment, leaves were badly wilted as indicated by a red box. (TIF 6252 kb) [file 12864_2019_5585_MOESM1_ESM.tif]

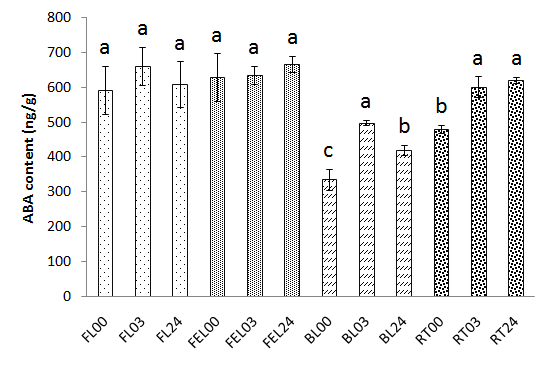

Supplement: Supplementary file 6 — Figure S2. ABA content determined in leaves and roots under drought. Data were shown as mean ± standard deviation derived from three biological replicates, and values with different letters were significant (P < 0.05) based on Duncan’s multiple range tests. (TIF 55 kb) [file 12864_2019_5585_MOESM6_ESM.tif]
